# Effect of Low-Level Laser Stimulation on EEG Power in Normal Subjects with Closed Eyes

**DOI:** 10.1155/2013/476565

**Published:** 2013-10-31

**Authors:** Jih-Huah Wu, Yang-Chyuan Chang

**Affiliations:** ^1^Department of Biomedical Engineering, Ming Chuan University, No. 5 Deming Road, Guishan Township, Taoyuan County 333, Taiwan; ^2^Department of Neurology, Min-Sheng General Hospital, No. 168 Jin-Kuo Road, Taoyuan City, Taoyuan County 330, Taiwan

## Abstract

In a previous study, we found that the low-level laser (LLL) stimulation at the palm with a frequency of 10 Hz was able to induce significant brain activation in normal subjects with opened eyes. However, the electroencephalography (EEG) changes to LLL stimulation in subjects with closed eyes have not been studied. In the present study, the laser array stimulator was applied to deliver insensible laser stimulations to the palm of the tested subjects with closed eyes (the laser group). The EEG activities before, during, and after the laser stimulation were collected. The EEG amplitude powers of each EEG frequency band at 19 locations were calculated. These power data were then analyzed by SPSS software using repeated-measure ANOVAs and appropriate posthoc tests. We found a pronounced decrease in the EEG power in alpha-bandwidth during laser simulation and then less decrease in the EEG power in delta-bandwidth in normal subjects with laser stimulation. The EEG power in beta-bandwidth in the right occipital area also decreased significantly in the laser group. We suggest that LLL stimulation might be conducive to falling into sleep in patients with sleep problems.

## 1. Introduction

The EEG activity can be affected by different stimulation modalities [1–8], including visual, auditory, and somatosensory stimulation. It has been proposed that, through the intermediary of adequate and effective electrocerebral modification (e.g., EEG activity), an appropriate sensory stimulation is able to induce a desired mental state, such as a relaxed or sleep state [9–12]. Such sensory stimulation can be applied to the fore or back of the head, scalp, eyes, nose root, temples, and also the specific acupoints. For example, Yasushim invented an apparatus to induce brain wave changes by an optically stimulating signals at a frequency close to actual human brain waves [9]. Siever designed a technique to activate the central nervous system (CNS) by auditory stimulations at different frequencies in the right or left brain hemisphere [10]. Sunnen proposed a method to generate sleep-inducing stimuli with a transducer [11]. Flagg et al. proposed an apparatus which can deliver a plurality of magnetic pulses from the nuchal region to influence the brain centers to obtain a desired mental state [12]. We reckon that low-level laser (LLL) stimulation should also be a good alternative to have the similar effect.

In our previous study, we found that the EEG activities of normal subjects with opened eyes could be affected by LLL stimulation at the left palm. With stimulation, the EEG powers in alpha- and theta-bandwidths in the posterior head regions increased, while the EEG power in beta-bandwidth in the frontal head regions decreased [13]. In that study, we required the tested subjects to keep eyes opened during the whole 30-minute-recording period, in order not to become drowsy or even fall into sleep. The aim of the present study is to investigate if there are any significant changes in EEG power to LLL stimulation in the normal subject with closed eyes. 

## 2. Subjects and Methods

Prior to the trial, the study protocol was approved by the Institutional Ethics Committee of Min-Sheng General Hospital. Each participant was required to give a written informed consent. This study was directed in conformity with the guidelines in the Helsinki Declaration. 

### 2.1. Participants

Twenty normal healthy subjects were included (mean age 21.0 ± 1.2 years, 14 males, 6 females) in the present study. Each subject received two trials: one trial with the laser stimulator being turned on (the laser group) and the other trial with the stimulator being not turned on (the control group). In the first trial, each subject was randomly and blindly assigned to either laser or control group. Several days later in the second trial, the tested subject was then arranged to enter into the laser or the control group, just different to that in his or her first trial. As we had to match the operation schedule of the examination room as well as the free time of the tested subjects, the intervals between two trials were not the same and ranged from 3 to 7 days.

Exclusion criteria included (a) having a history of psychiatric disorders, for example, major depression, substance abuse, schizophrenic, or paranoid disorder, (b) having cardiopulmonary disease, and (c) receiving medication currently.

### 2.2. Laser

In this study, the same laser stimulator in our previous study was used [13]. The LAS consists of 6 laser diodes (LDs). Each LD was set at 7 mW output for minimum stimulation in this study, and the operational frequency was set at 10 Hz, duty cycle 50%. The light of laser diode without any collimated lens was a stripe shape due to the different divergence angle in horizontal (10°) and vertical (30°) directions. The area of the laser light was approximately equal to 14.8 mm^2^ at 10 mm distance. Thus, the dosage would be approximately 20 joules/cm^2^ for 10 minutes treatment; it is insensible on operation. 

### 2.3. Procedure

The “double-blind randomized trial” was used in this study. In each trial, the subjects did not know which group they were in. The subject sat in an armchair and was then required to put the left palm on the LAS device. He or she was instructed to relax, follow the eyes-closed directive, and withhold any movements. In the laser group, the laser diodes were turned on for 10 minutes and not turned on in the control group. In the beginning, each subject was required to relax for five minutes in order to be in a stale physiological state. The ongoing EEG was recorded with closed eyes in three stages (6 sessions): before stimulation (baseline 5 min, session 1), during stimulation (laser stimulation, 10 min, session 2 and session 3), after stimulation (poststimulation, 15 min, and session 4, 5, and 6). This procedure was similar to that in our previous study [13], but the patients kept their eyes closed. The EEG technologist was required not to disturb the tested subject even evidence of drowsiness or sleep emerged out of the ongoing EEG. 

### 2.4. Control

The low-level infrared laser diode is invisible and emits no heat or any other detectable indication; therefore, it is ideal for a double-blind study. When the subjects received a sham laser stimulation in the control group, they underwent the same procedure as in the laser group, but the laser stimulator was not turned on. 

### 2.5. EEG Recording and Measurement

During experiment, the tester was required to put his or her left palm on the LAS. An electroencephalograph (Neurofax model EEG-1000, NIHON KOHDEN) was used in this study. The band pass was set at 0.5–70.0 Hz. The variation of EEG potential was recorded on the scalp with Ag/AgCl recording electrodes. Electrode placement was arranged following international 10–20 system. A quantitative referential (monopolar montage) EEG was recorded with 19 electrodes with linked earlobe references. The sampling rate was set at 256 samples per second. An ECG was recorded by placing electrodes on both hands. EEG data were analyzed to provide power data for the 19 recording locations in each of the four bandwidths (delta, 0.5–3.5 Hz; theta, 4–7 Hz; alpha, 8–13 Hz; beta, 13–50 Hz) with the software Neurofax version 05–80. The mean and standard deviation of calculated values were expressed as “Mean ± SD”.

### 2.6. Statistical Analysis

A one-way repeated-measure ANOVA and appropriate posthoc tests were used to compare the differences of EEG band power before and after LAS stimulation from 19 recording locations in each of four band passes. Two-tailed paired *t*-test was applied to compare the difference of EEG band power before and after LAS stimulation from F4, C4, P4, O2, F3, C3, P3, and O1 electrodes. All the statistical analyses were executed with SPSS software (version 11). A statistical significance was recognized as *P* value <0.05. 

## 3. Result

**Figure 1:**
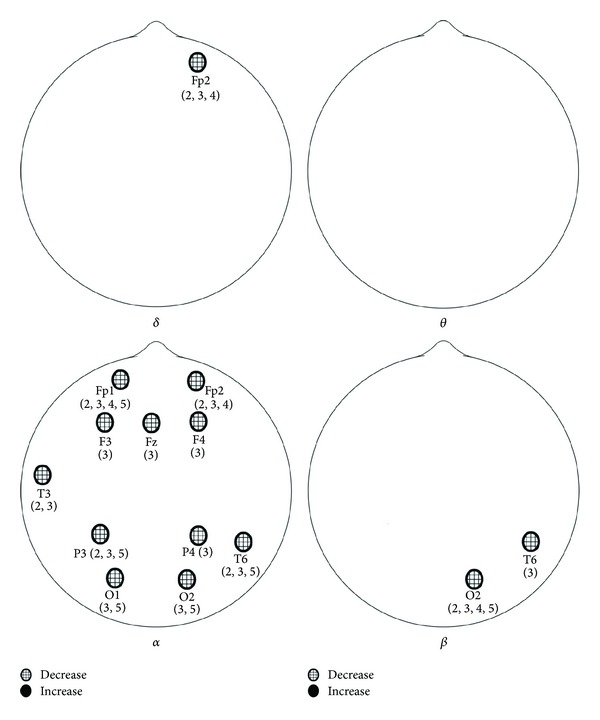
Significant changes from baseline EEG activity during 10 Hz stimulation. Numbers indicate time period of significance (1 first 5 min of baseline, 2 first 5 min of LAS, 3 second 5 min of LAS, 4 fourth 5 min, 5 fifth 5 min, and 6 last 5 min).

**Figure 2:**
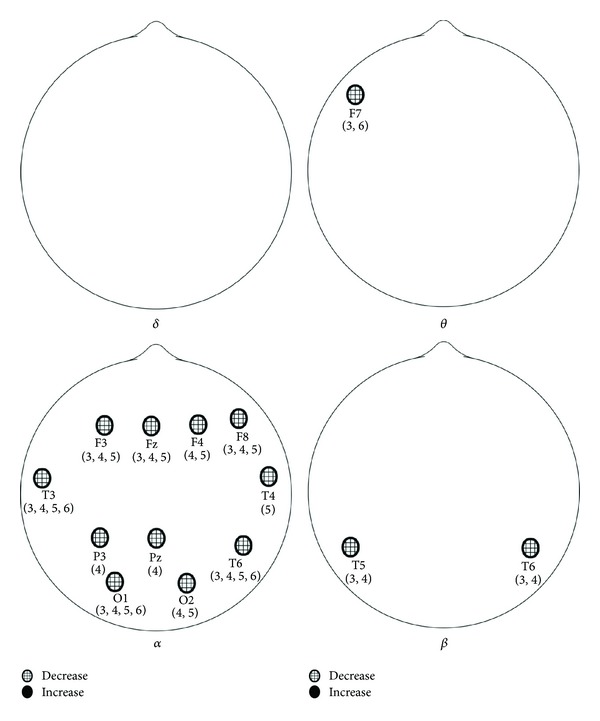
Significant changes from baseline EEG activity in placebo group. Numbers indicate time period of significance (1 first 5 min of baseline, 2 first 5 min of LAS, 3 second 5 min of LAS, 4 fourth 5 min, 5 fifth 5 min, and 6 last 5 min).

For the laser group, the ANOVA analysis indicated a total of 14 significant locations with *P* < 0.05. The locations, by band pass, were as follows: delta: Fp2, alpha: Fp1, Fp2, F3, Fz, F4, T3, P3, P4, T6, O1, and O2; beta: T6, O2; theta: no significant ANOVA results. For the placebo group, the ANOVA analysis indicated a total of 14 significant locations with *P* < 0.05 too but in different places. The locations, by band pass, were as follows: delta: no significant ANOVA results, theta: F7, alpha: F3, Fz, F4, F8, T3, T4, P3, Pz, T6, O1, and O2; beta: T5, T6. Locations indicating significant changes from baseline power during and after the 10 min LAS stimulation session are presented in Figure 1 for the laser group and in Figure 2 for the placebo group. Even the differences between these two groups are not significant at all recording locations, but it is worthy to mention that the alpha power decreased significantly in frontal regions (Fp1 and Fp2) and the beta power decreased significantly in occipital lobe (O2) in the laser group. 

**Figure 3:**
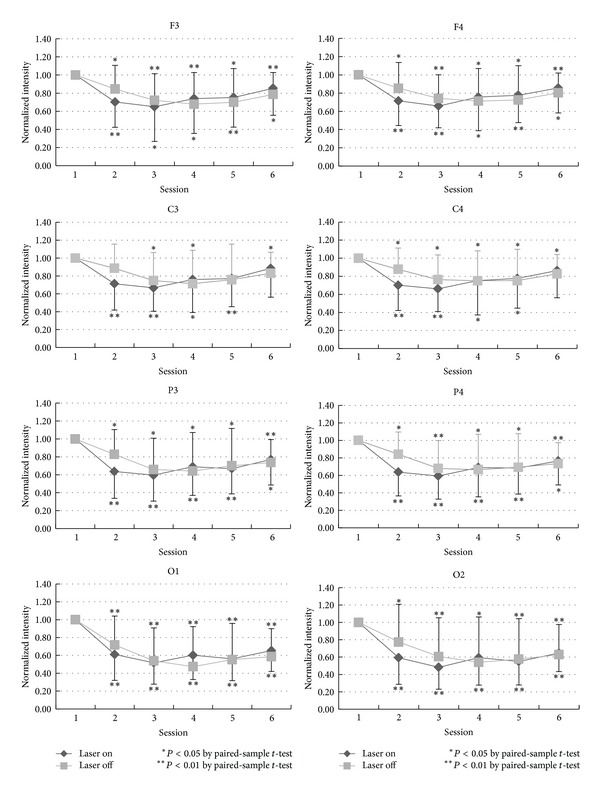
The statistical analysis of the alpha-band by comparing the baseline and each session in laser and placebo group is shown in different locations: F3, C3, P3, O1, F4, C4, P4, and O2.

The ROI (regions of interest: frontal, central, parietal, and occipital regions) were analyzed with paired *t*-test analysis.  Figure 3 shows the temporal change of the normalized intensity of EEG power in alpha-bandwidth in the six consecutive sessions in the laser (rhomboid spot) and the control (square spot) group. In this figure, the normalized intensity in the ordinate was obtained from division of the measured alpha power by the corresponding alpha power in the first session. In either laser or control group, the alpha power significantly decreased from session 2 to session 6, especially in the posterior head region (i.e., P3, P4, O1, and O2). The decrease in alpha power in sessions 2 and 3 is more prominent in the laser group than in the control group. 

**Figure 4:**
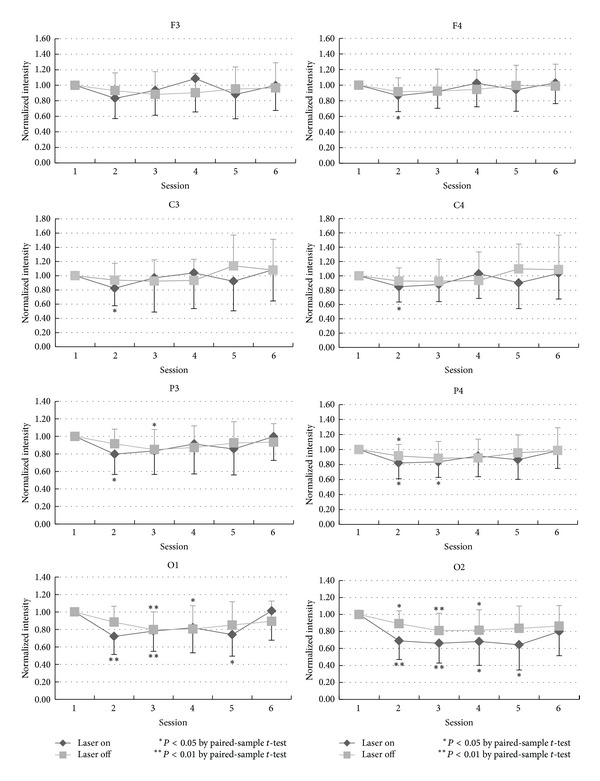
The statistical analysis of the beta-band by comparing the baseline and each session in laser and placebo group is shown in different locations: F3, C3, P3, O1, F4, C4, P4, and O2.

The temporal changes in the normalized intensity of EEG power in beta-bandwidth are showed in Figure 4. In either laser or control group, there was a variation in beta power in the anterior head area (F3, F4, C3, and C4) from session 2 to session 6, but a tendency to decrease in beta power was seen. A decrease of beta power was seen in the posterior head region, especially the occipital area (O1 and O2). In the right occipital area, decrease in beta power in the laser group was greater than that in the control group, and it has significant meaning in session 2, *P* < 0.05.

**Figure 5:**
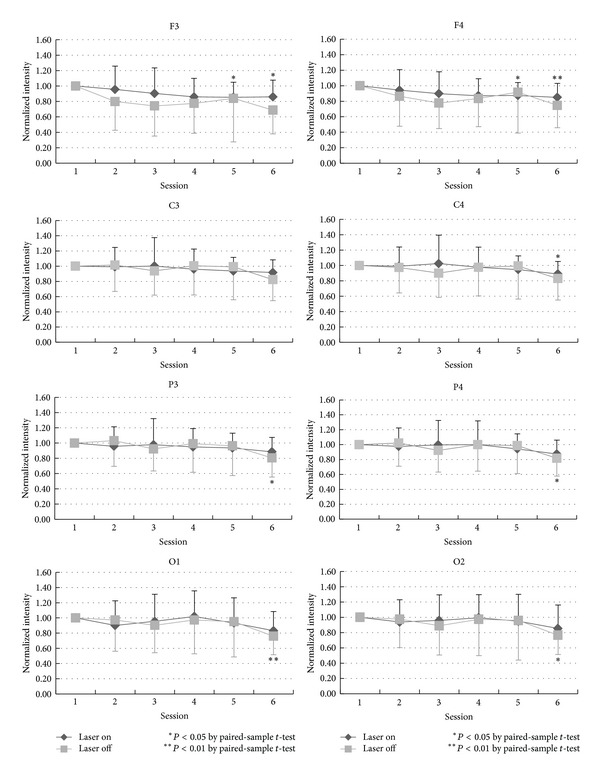
The statistical analysis of the theta-band by comparing the baseline and each session in laser and placebo group is shown in different locations: F3, C3, P3, O1, F4, C4, P4, and O2.

Figure 5 shows the temporal changes in the normalized intensity of EEG power in theta-bandwidth. As compared with that in the first session, little changes in theta power were found. There were only mild decrease in the frontal area (F3 and F4) and mild decrease in the last session in nearly all the head areas. Difference in intensity changes between two groups was minimal and not significant. 

**Figure 6:**
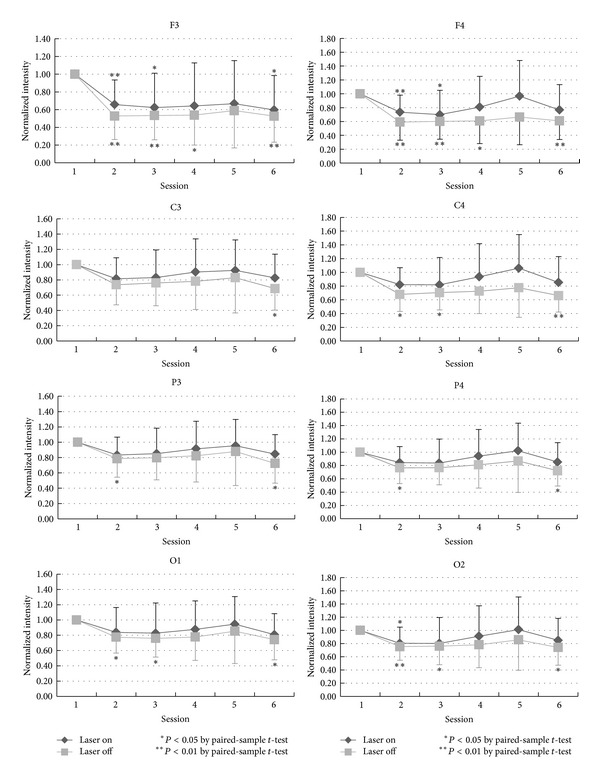
The statistical analysis of the delta-band by comparing the baseline and each session in laser and placebo group is shown in different locations: F3, C3, P3, O1, F4, C4, P4, and O2.

The temporal changes in the normalized intensity of EEG power in delta-bandwidth are showed in Figure 6. A similar pattern of temporal changes in the delta power was found in all of the head areas; that is, the delta power decreased in the 2nd and 3rd sessions and gradually returned toward the original level (the first session) in the 4th and 5th sessions, with an exception of delta power in the frontal area (F3 and F4). The delta power dropped again in the last session. Degree of drop in the delta power in some areas was greater in the control than in the laser group.

## 4. Discussion

 When taking routine awake EEG in the EEG laboratory, the patient is instructed to sit quietly, to relax himself, and to keep eyes closed. In such a circumstance, the patient not surprisingly easily becomes drowsy or even falls into sleep. Therefore, the EEG technologist has to watch the ongoing EEG tracings carefully and to awake the patient if EEG evidence of drowsiness appears. In our previous study, we required the tested subjects to keep eyes opened and found LLL stimulation able to induce brain activation in normal awake subjects. In the present study, we let the tested subjects close their eyes and relax completely. We did not prevent them from becoming drowsy or falling into sleep. Electroencephalographically, almost all the tested subjects could not maintain awake during the whole experiment period.

 In normal, awake, relaxed adults with closed eyes, the dominant brain waves are alpha rhythms distributed mainly in the posterior head region. The alpha rhythms are attenuated by visual attention and mental efforts [14]. The typical EEG changes in drowsiness in normal adults are gradual or brisk dropout of alpha rhythms, appearance of desynchronized low-voltage slow waves (2–7 Hz), and emergence of vertex sharp waves. Anterior diffusion of alpha rhythms and increased beta activities (mainly 18–25 Hz) in the frontocentral areas are occasionally noted. Then, sleep spindles, vertex sharp waves, and K-complexes may probably emerge from a background with low amplitude and mixed frequency. High-voltage rhythmic theta or delta waves are very rare [15]. 

 Curves in Figure 3 to Figure 6, which showed the temporal changes of EEG power in four different bandwidths, reliably reflect the awake-drowsy-sleepy state of the tested subjects. In a restful situation and a relaxed body/mind, they almost unavoidably entered a drowsy and sleepy state. Dropout of alpha rhythms in drowsiness can result in a decreased EEG power in alpha-bandwidth and the degree of decrease is reasonably more prominent in the posterior head region (Figure 3). The rebound of EEG power in alpha-bandwidth to some extent in the latter sessions (sessions 5 and 6) may be related to anterior diffusion of alpha rhythms in drowsiness and emergence of sleep spindles (12–14 Hz, part of them being in the alpha range) in the light sleep. 

 Similar to other EEG activities, beta waves in drowsiness also become less prominent. The temporal curve of EEG power in beta-bandwidth therefore decreased in the earlier sessions (Figure 4). However, occasional enhancement of beta activity in light sleep is the most likely reason to cause the normalized intensity of EEG power in beta-bandwidth undulated gently around the original level. 

In awake adults, theta waves are abnormal if occurring excessively. However, desynchronized low-voltage theta waves are normally seen in drowsiness or sleep. The appearance of theta waves is one important hallmark of onset of drowsiness [16]. In this study, there were no significant changes in EEG power in theta-bandwidth (Figure 5). All of the tested subjects were university students and nearly all of them were used to stay up late. Hence, sitting in a comfortable armchair with closed eyes and doing nothing, almost all of the tested subjects drowsed even early in the first session in EEG recording. The EEG power in theta-bandwidth therefore was maintained rather stable in the whole recording periods (Figure 5). A mild drop of the normalized intensity in the last session (session 6) is of uncertain significance. The reason for decreased theta power in the frontal region (F3 and F4) is considered similar to that for EEG power in delta-bandwidth (see below).

 In visual analysis of EEG recording, delta waves are not seen in the normal awake adults. They are the main EEG activities in deep sleep. With power spectrum analysis, EEG power in the delta-bandwidth is present, probably related to slow waves subharmonic to alpha or other rhythms. Artifacts arising from blinking or eyeball movements usually resemble EEG waves in the delta (sometimes theta) range, especially in the anterior head region. Under power spectrum analysis, they are not possibly differentiated from genuine EEG delta waves and they also play an important role in EEG power in delta-bandwidth (and also theta). The slow-rolling eye movements in light sleep or slow-wave sleep did not contribute significantly to the delta power in the present study, for their frequency is usually below 0.5 Hz and outside the range of spectrum analysis. Temporal changes in EEG power in delta-bandwidth (Figure 6) are compatible with appearance of desynchronized low-voltage slow waves in drowsiness or light sleep. In the frontal areas (F3, F4), more prominent drop in delta power is considered caused by less or no eyeball movement artifacts in drowsiness or light sleep. Similar to the theta power, there is also a mild drop of the normalized intensity in the last session (session 6) of uncertain significance.

 Main differences in EEG power in different bandwidths between the laser and the control group include more prominent decreased EEG power in the alpha-bandwidth during LLL stimulation (sessions 2 and 3 in the laser group), more decrease in the beta-bandwidth at O2 in the laser group, and less decrease in the delta-bandwidth in the latter sessions in the laser group especially in the right hemisphere. Although the actual and detailed mechanisms of LLL-induced physiological changes in the brain are not well known, on the basis of sensory physiology, we deem that such effects should be more pronounced in the hemisphere contralateral to side of stimulation, that is, the right hemisphere in the present study. Side-to-side differences in LLL-induced change in the EEG power in the present study (Figure 3 to Figure 6) are considered related to laser stimulation at the left palm. 

 Transcranial electric stimulation to evoke generalized convulsions (electroconvulsive therapy) is a well-known technique to treat schizophrenia and depression. Transcranial magnetic stimulation has been tried for the treatment of major depression [17]. In addition to direct brain stimulation, stimulation at the peripheral nerve has also been applied to the treatment of some neurological or psychiatric diseases, such as vagus nerve stimulation in intractable epilepsy and resistant depression [18, 19] and occipital nerve stimulation for cluster headache and other types of headache [20, 21]. We consider LLL stimulation in the present study to be comparable with a kind of peripheral nerve stimulation. 

In view of more decrease in the alpha power during LLL stimulation (earlier sessions in the present study), we infer that LLL stimulation is helpful in sleep induction. According to less decrease in the delta power in the latter sessions in the laser group, we postulate that LLL stimulation can lead to a deeper drowsiness or sleep state. So, we suggest that LLL stimulation can be one of the nonpharmacological solutions for patients with sleep problems. However, further studies are necessary.

## 5. Conclusion

The effects of low-level laser stimulation on the EEG power in normal subjects with closed eyes were investigated. Nearly all the tested subjects were found to fall into drowsiness or light sleep easily. Pronounced decrease in the EEG power in alpha-bandwidth during laser simulation and then less decrease in the delta power were found in normal subjects with laser stimulation. We suggest that low-level laser stimulation is probably useful for patients with sleep problems.
